# Designing Artificial Neural Networks Using Particle Swarm Optimization Algorithms

**DOI:** 10.1155/2015/369298

**Published:** 2015-06-29

**Authors:** Beatriz A. Garro, Roberto A. Vázquez

**Affiliations:** ^1^Instituto en Investigaciones en Matemáticas Aplicadas y en Sistemas, Universidad Nacional Autónoma de México, Ciudad Universitaria, 04510 Mexico City, DF, Mexico; ^2^Intelligent Systems Group, Faculty of Engineering, La Salle University, Benjamín Franklin 47, Colonia Condesa, 06140 Mexico City, DF, Mexico

## Abstract

Artificial Neural Network (ANN) design is a complex task because its performance depends on the architecture, the selected transfer function, and the learning algorithm used to train the set of synaptic weights. In this paper we present a methodology that automatically designs an ANN using particle swarm optimization algorithms such as Basic Particle Swarm Optimization (PSO), Second Generation of Particle Swarm Optimization (SGPSO), and a New Model of PSO called NMPSO. The aim of these algorithms is to evolve, at the same time, the three principal components of an ANN: the set of synaptic weights, the connections or architecture, and the transfer functions for each neuron. Eight different fitness functions were proposed to evaluate the fitness of each solution and find the best design. These functions are based on the mean square error (MSE) and the classification error (CER) and implement a strategy to avoid overtraining and to reduce the number of connections in the ANN. In addition, the ANN designed with the proposed methodology is compared with those designed manually using the well-known Back-Propagation and Levenberg-Marquardt Learning Algorithms. Finally, the accuracy of the method is tested with different nonlinear pattern classification problems.

## 1. Introduction

Artificial Neural Networks (ANNs) are system composed of neurons organized in input, output, and hidden layers. The neurons are connected to each other by a set of synaptic weights. An ANN is a powerful tool that has been applied in a broad range of problems such as pattern recognition, forecasting, and regression. During the learning process, the ANN continuously changes their synaptic values until the acquired knowledge is sufficient (until a specific number of iterations is reached or until a goal error value is achieved). When the learning process or the training stage has finished, it is mandatory to evaluate the generalization capabilities of the ANN using samples of the problem, different to those used during the training stage. Finally, it is expected that the ANN can classify with an acceptable accuracy the patterns from a particular problem during the training and testing stage.

Several classic algorithms to train an ANN have been proposed and developed in the last years. However, many of them can stay trapped in nondesirable solutions; that is, they will be far from the optimum or the best solution. Moreover, most of these algorithms cannot explore multimodal and noncontinuous surfaces. Therefore, other kinds of techniques, such as bioinspired algorithms (BIAs), are necessary for training an ANN.

BIAs have a good acceptance by the Artificial Intelligence community because they are powerful optimization tools and can solve very complex optimization problems. For a given problem, BIAs can explore big multimodal and noncontinuous search spaces and can find the best solution, near the optimum value. BIAs are based on nature's behavior described as* swarm intelligence*. This concept is defined in [1] as a property of systems composed of unintelligent agents with limited individual capabilities but with an intelligent collective behavior.

There are several works that use evolutionary and bioinspired algorithms to train ANN as another fundamental form of learning [2]. Metaheuristic methods for training neural networks are based on local search, population methods, and others such as cooperative coevolutionary models [3].

An excellent work where the authors show an extensive literature review of evolutionary algorithms that are used to evolve ANN is [2]. However, most of the reported researches are focused only on the evolution of the synaptic weights, parameters [4], or involve the evolution of the neuron's numbers for hidden layers, but the number of hidden layers is established previously by the designer. Moreover, the researches do not involve the evolution of transfer functions, which are an important element of an ANN that determines the output of each neuron.

For example, in [5], the authors proposed a method that combines Ant Colony Optimization (ACO) to find a particular architecture (the connections) for an ANN and Particle Swarm Optimization (PSO) to adjust the synaptic weights. Other researches like [6] implemented a modification of PSO mixed with Simulated Annealing (SA) to obtain a set of synaptic weights and ANN thresholds. In [7], the authors use Evolutionary Programming to get the architecture and the set of weights with the aim to solve classification and prediction problems. Another example is [8] where Genetic Programming is used to obtain graphs that represent different topologies. In [9], the Differential Evolution (DE) algorithm was applied to design an ANN to solve a weather forecasting problem. In [10], the authors use a PSO algorithm to adjust the synaptic weights to model the daily rainfall-runoff relationship in Malaysia. In [11], the authors compare the back-propagation method versus basic PSO to adjust only the synaptic weights of an ANN for solving classification problems. In [12], the set of weights are evolved using the Differential Evolution and basic PSO.

In other works like [13], the three principle elements of an ANN are evolved at the same time: architecture, transfer functions, and synaptic weights. The authors proposed a New Model of a PSO (NMPSO) algorithm, while, in [14], the authors solve the same problem by means of a Differential Evolution (DE) algorithm. Another example is [15], where the authors used an Artificial Bee Colony (ABC) algorithm to evolve the design of an ANN with two different fitness functions.

This research has significant contributions in comparison with these last three works. First of all, eight fitness functions are proposed to deal with three common problems that emerge during the design of the ANN: accuracy, overfitting, and reduction of the ANN. In that sense, to handle better the problems that emerge during the design of the ANN, the fitness functions take into account the classification error, mean square error, validation error, reduction of architectures, and a combination of them. Furthermore, this research explores the behavior of three bioinspired algorithms using different values for their parameters. During the experimentation phase, the best parameter's values for these algorithms are determined to obtain the best results. In addition, the best configuration is used to generated a set of statistically valid experiments for each selected classification problem. Moreover, the results obtained with the proposed methodology in terms of the connection's number, the neuron's number, and the transfer functions selected for each ANN are presented and discussed. Another contribution of this research is related to a new metric that allows comparing efficiently the results provided by an ANN generated with the proposed methodology. This metric takes into account the recognition rate obtained during training and testing stages where testing accuracy is more weighted in comparison to training accuracy. Finally, the results achieved by the three bioinspired algorithms are compared against those achieved with two classic learning algorithms. The selection of the three bioinspired algorithms was done because NMPSO is a relatively new algorithm (proposed in 2009) which is based on the metaphor of basic PSO technique so it is important to compare its performance with others inspired in the same phenomenon.

In general, it is possible to define the problem to be solved as giving a set of input patterns *X* = {**x** ^1^,…, **x** ^*p*^}, **x** ∈ *ℝ* ^*n*^, and a set of desired patterns *D* = {**d** ^1^,…, **d** ^*p*^}, **d** ∈ *ℝ* ^*m*^, and finding the ANN represented by *W* ∈ *ℝ* ^*q*×(*q* + 3)^ such that a function defined by min(*F*(*D*, *X*, *W*)) is minimized and *q* defined the maximum number of neurons. It is important to remark that the search space involves three different domains (architecture, synaptic weight, and transfer functions).

This research provides a complete study about how an ANN can be automatically designed by applying bioinspired algorithms, particularly using the Basic Particle Swarm Optimization (PSO), Second Generation PSO (SGPSO), and New Model of PSO (NMPSO). The proposed methodology evolves at the same time the architecture, the synaptic weights, and the kind of transfer functions in order to design the ANNs that provide the best accuracy for a particular problem. Moreover, a comparison of the Particle Swarm algorithm performance versus classic learning methods (back-propagation and Levenberg-Marquardt) is presented. In addition, in this research is presented a new way to select the maximum number of neurons (MNN). The accuracy of the proposed methodology is tested solving some real and synthetic pattern recognition problems. In this paper, we show the results obtained with ten classification problems of different complexities.

The basic concepts concerning the three PSO algorithms and ANN are presented in Sections 2 and 3, respectively. In Section 4 the methodology and the strategy used to design the ANN automatically are described. In Section 5 the eight fitness functions used in this research are described. In Section 6, the experimental results about tuning the parameters for PSO algorithms are described. Moreover, the experimental results are outlined in Section 7. Finally, in Sections 8 and 9 the general discussion and conclusions of this research are given.

## 2. Particle Swarm Optimization Algorithms

In this section, three different algorithms based on PSO metaphor are described. The first one is the original PSO algorithm. Then, two algorithms which improve the original PSO are shown: the Second Generation of PSO and a New Model of PSO.

### 2.1. Original Particle Swarm Optimization Algorithm

The Particle Swarm Optimization (PSO) algorithm is a method for the optimization of continuous nonlinear functions proposed by Eberhart et al. [16]. This algorithm is inspired by observations of social and collective behavior on the movements of bird flocks in search of food or survival as well as fish schooling. A PSO algorithm is inspired on the movements of the best member of the population and at the same time also on their own experience. The metaphor indicates that a set of solutions is moving in a search space with the aim to achieve the best position or solution.

The population is considered as a cumulus of particles *i* where each represents a position **x**_*i*_ ∈ *ℝ* ^*D*^, *i* = 1,…, *M* in a multidimensional space. These particles are evaluated in a particular optimization function to recognize their fitness value and save the best solution. All the particles change their position in the search space according to a velocity function **v**_*i*_ which takes into account the best position of a particle in a population **p**_*g*_ ∈ *ℝ* ^*D*^ (i.e., social component) as well as their own best position **p**_*i*_ ∈ *ℝ* ^*D*^ (i.e., cognitive component). The particles will move in each iteration to a different position until they reach an optimum position. At each time *t*, the particle velocity *i* is updated using

(1) vit+1=ωvit+c1r1pit−xit+c2r2pgt−xit,

where *ω* is the inertia weight and typically set up to vary linearly from 1 to 0 during the course of an iteration run; *c*_1_ and *c*_2_ are acceleration coefficients; *r*_1_ and *r*_2_ are uniformly distributed random numbers between (0,1). The velocity **v**_*i*_ is limited to the range [*v*_max_, *v*_min_]. Updating velocity in this way enables the particle *i* to search for its best individual position **p**_*i*_(*t*), and the best global particle position *i* is computed as in

(2) $$\begin{array}{c} x_{i}\left(\;t+1\;\right)=x_{i}\left(\;t\;\right)+v_{i}\left(\;t+1\;\right). \end{array}$$

### 2.2. Second Generation of PSO Algorithm

The SGPSO algorithm [17] is an improvement of the original PSO algorithm that considers three aspects: the local optimum solution of each particle, the global best solution, and a new concept, the geometric center of optimum swarm. The authors explain that the birds keep a certain distance from the swarm center (food). On the other hand, no bird accurately calculates the position of the swarm center every time. Bird flocking always stays in the same area for a specified time, during which the swarm center will be kept fixed in every bird eyes. Afterward, the swarm moves to a new area. Then all birds must keep a certain distance in the new swarm center. This fact is the basis of the SGPSO.

The position of the geometric centre $\bar{P}\in {\mathbb{R}}^{D}$ of the optimum swarm is updated according to

(3) $$\begin{array}{c} \bar{P}=\frac{1}{M}\overset{M}{\underset{i=1}{\sum }}p_{i},\;\text{if CI mod }T=0, \end{array}$$

where *M* is the number of particles in the swarm, CI is the current iteration number, and *T* is the geometric centre updating time of optimum swarm with a value between [1, MAXITER].

In SGPSO the velocity is updated by (4) and the position of each particle by (5):

(4) vit+1=ωvit+c1r1pit−xit+c2r2pgt−xit+c3r3P¯−xit,

(5) xit+1=xit+vit+1,

where *c*_1_, *c*_2_, and *c*_3_ are constants called acceleration coefficients, *r*_1_, *r*_2_, and *r*_3_ are random numbers in the range [0,1], and *w* is the velocity inertia.

### 2.3. New Model of Particle Swarm Optimization

This algorithm was proposed by Garro et al. [13] and is based on some ideas that other authors proposed to improve the basic PSO algorithm [4]. These ideas are described in next paragraphs.

Shi and Eberhart [18] proposed a linearly varying inertia weight over the course of generations, which significantly improves the performance of Basic PSO. The following equation shows us how to compute the inertia:

(6) $$\begin{array}{c} w=\left(\;w_{1}-w_{2}\;\right)\times \frac{\text{MAXITER}-\text{iter}}{\text{MAXITER}}+w_{2}, \end{array}$$

where *w*_1_ and *w*_2_ are the initial and final values of the inertia weight, respectively, iter is the current iteration number, and MAXITER is the maximum number of allowable iterations. The empirical studies in [18] indicated that the optimal solution could be improved by varying the value of *w* from 0.9 at the beginning of the evolutionary process to 0.4 at the end of the evolutionary process.

Yu et al. [4] developed a strategy that when the global best position is not improving with the increasing number of generations, each particle *i* will be selected by a predefined probability from the population, and then a random perturbation is added to each velocity vector dimension **v**_*i*_ of the selected particle *i*. The velocity resetting is computed as in

(7) $$\begin{array}{c} v_{i}=v_{i}+\left(\;2\times r-1\;\right)\times v_{\max}, \end{array}$$

where *r* is a uniformly distributed random number in the range (0,1) and *v*_max_ is the maximum random perturbation magnitude to each selected particle dimension.

Based on some evolutionary schemes of Genetic Algorithms (GA), several effective mutation and crossover operators have been proposed for PSO. Løvberg et al. [19] proposed a crossover operator in terms of a certain crossover rate *α* defined in

(8) $$\begin{array}{c} ch_{1}\left(\;x_{i}\;\right)=r_{i}\;{\text{par}}_{1}\left(\;x_{i}\;\right)+\left(\;1-r_{i}\;\right){\text{par}}_{2}\left(\;x_{i}\;\right), \end{array}$$

where *r*_*i*_ is a uniformly distributed random number in the range (0,1), ch_1_ is the offspring, and par_1_ and par_2_ are the two parents randomly selected from the population.

The offspring velocity is calculated in the following equation as the sum of the two parents velocity vectors, normalized to the original length of each parent velocity vector:

(9) $$\begin{array}{c} ch_{1}\left(\;v_{i}\;\right)=\frac{{\text{par}}_{1}\left(v_{i}\right)+{\text{par}}_{2}\left(v_{i}\right)}{\left|{\text{par}}_{1}\left(v_{i}\right)+{\text{par}}_{2}\left(v_{i}\right)\right|}\left|\;{\text{par}}_{1}\left(\;v_{i}\;\right)\;\right|. \end{array}$$

Higashi and Iba [20] proposed a Gaussian mutation operator to improve the performance of PSO in terms of a certain mutation rate *β* defined in

(10) $$\begin{array}{c} ch\left(\;x_{i}\;\right)=\text{par}\left(\;x_{i}\;\right)+\frac{\text{MAXITER}-\text{iter}}{\text{MAXITER}}N\left(\;0,1\;\right), \end{array}$$

where ch is the offspring, par is the parent randomly selected from the population, iter is the current iteration number and MAXITER is the maximum number of allowable iterations, and *N* is a Gaussian distribution. Utilization of these operators in PSO has the potential to achieve faster convergence and find better solutions.

Mohais et al. [6, 21] used random neighborhoods in PSO, together with dynamism operators.

In the NMPSO, the use of dynamic random neighborhoods that change in terms of certain rates *γ* is proposed. First of all, a maximum number of neighborhoods MAXNEIGH is defined in terms of population size divided by 4. With this condition at least each neighborhood *K*_*n*_, *n* = 1,…, MAXNEIGH, will have 4 members. Then, the members of each neighborhood *K*_*n*_ are randomly selected, and the best particle **p**_*g*_*K*_*n*___ is computed. Finally, the velocity of each particle *i* is updated as in

(11) vit+1=ωvit+c1r1pit−xit+c2r2pgKnt−xt,

for all *i* ∈ *K*_*n*_, *n* = 1,…, MAXNEIGH.

The NMPSO combines the varying schemes of inertia weight *ω* and acceleration coefficients *c*_1_ and *c*_1_, velocity resetting, crossover and mutation operators, and dynamic random neighbourhoods [13]. The NMPSO algorithm is described in Algorithm 1.

## 3. Artificial Neural Networks

An ANN is a system that performs a mapping between input and output patterns that represent a problem [22]. The ANNs learn information during the training process after several iterations. When the learning process finishes, the ANN is ready to classify new information, predict new behaviours, or estimate nonlinear function problems. Its structure consists of a set of neurons (represented by functions) connected among others organized in layers. The patterns that codify the real problem codification **a** ∈ *ℝ* ^*N*^ are sent through layers and the information is transformed with the corresponding synaptic weights **W** ∈ *ℝ* ^*N*^ (values between 0 and 1). Then, neurons in the following layers perform a summation of this information depending on whether there exists a connection between them. In addition, in this summation another input called bias is considered where the value of its input is 1. This bias is a threshold that represents the minimum level that a neuron needs for activating and is represented by *θ*. The summation function is presented in

(12) $$\begin{array}{c} o=\overset{N}{\underset{i=1}{\sum }}a_{i}w_{i}+\theta . \end{array}$$

After that, the result of the summation is evaluated in transfer functions *f*(*o*) activated by the neuron input. The result is the output neuron, and this information is sent to the other connected neurons until they reach the last layer. Finally, the output of the ANN is obtained.

The learning process consists of adapting the synaptic weights until they reach the desire behaviour. The output is evaluated to measure the performance of the ANN; if the output is not as desired, the synaptic weights have to be changed or adjusted in terms of the input patterns **a** ∈ *ℝ* ^*N*^. There are two ways to verify if the ANN has learned: first, the ANN computes grades similarity between input patterns and information that it knew before (nonsupervised learning). Secondly, the ANN output with desire patterns **y** ∈ *ℝ* ^*M*^ is compared (supervised learning). In our case, supervised learning where the objective is to produce an output approximation with the desired patterns of a input-output samples set *p* is applied (see the following equation):

(13) $$\begin{array}{c} T^{\xi }=\left\{\;\left(\;a^{\xi }\in R^{N},\;\;d^{\xi }\in R^{M}\;\right)\;\right\}\;\forall \xi =1,\ldots ,p, \end{array}$$

where **a** is the input pattern and **d** the desired response.

Given the training sample **T** ^*ξ*^, the requirement is to design and compute the neural network free parameters so that the actual output **y** ^*ξ*^ of the neural network due to **a** ^*ξ*^ is close enough to **d** ^*ξ*^ for all *ξ* in a statistical sense [15]. We may use the mean square error (MSE) given by (14) as the first objective function to be minimized. There are algorithms that adjust the synaptic weights to obtain a minimum error such as the classic back-propagation (BP) algorithm [23, 24]. This algorithm like others is based on the descendant gradient technique, which can stay trapped in a local minimum. Furthermore, a BP algorithm cannot solve noncontinuous problems. For this reason, the applications of other techniques that can solve noncontinuous and nonlinear problems are necessary to implement for obtaining a better performance of the ANN and solving really complex problems:

(14) $$\begin{array}{c} e=\frac{1}{p\cdot M}\overset{p}{\underset{\xi =1}{\sum }}\;\overset{M}{\underset{i=1}{\sum }}{\left(\;d_{i}^{\xi }-y_{i}^{\xi }\;\right)}^{2}. \end{array}$$

## 4. Proposed Methodology

The most important elements to design and improve the accuracy of an ANN are the architecture (or topology), the set of transfer functions (TF), and the set of synaptic weights and bias. These elements should be codified into the individual that represents the solution of our problem. The solutions generated by the bioinspired algorithms will be measured by the fitness function with the aim to select the best individual which represents the best ANN. The three bioinspired algorithms (basic PSO, SGPSO, and NMPSO) are going to lead the evolutionary learning process until finding the best ANN by using one of the eight fitness functions proposed in this paper. It is important to remark that only pattern classification problems will be solved by the proposed methodology.

The methodology is evaluated with three particle swarm algorithms and eight fitness functions. Therefore, this involves an extensive behavioral study for each algorithm. Another point to review is the maximum number of neurons (MNN) used by the methodology to generate the ANN which is directly related to the dimension of the individual. Due to the information needed to determine the size of the individuals for a specific problem only depending on the input and output patterns (because the supervised learning is applied), it was necessary to propose an equation that allow us to obtain the MNN to design the ANN. This equation is explained in the individual section.

**Figure 1:**
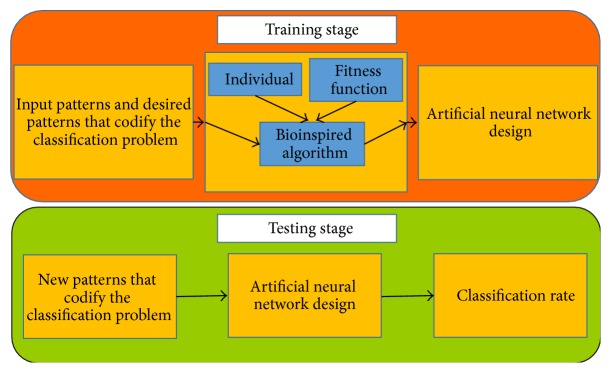
General diagram of the proposed methodology.

In Figure 1, a diagram of the proposed methodology is shown. During the training stage, it is necessary to define the individual and the fitness functions to evaluate each individual. The size of the individual depends on the size of the input patterns as well as the desire patterns. The individual will be evolved during a certain time to obtain the best solution (with a minimum error). At the end of the learning process, it is expected that the ANN provides an acceptable accuracy during the training and testing stage.

### 4.1. Individual

When solving an optimization problem, the problem has to be described as a feasible model. After the model is defined, the next step is focused on designing the individual that codifies the solution for the problem. Equation (15) shows an individual represented with a matrix that codifies the ANN design. This codification was previously described in [13–15]. As it is necessary to evolve the three ANN elements at the same time, a matrix **W** ∈ *ℝ* ^*q*×(*q* + 3)^ is composed by three principal parts with the following information: first, the topology (*T*), second the synaptic weights and bias (SW), and third the transfer functions (TF), where *q* is the maximum number of neurons (MNN) defined by *q* = *m* + *n* + ((*m* + *n*)/2), *n* is the input patterns vector dimension, and *m* is the desired patterns vector dimension:

(15) $$\begin{array}{c} \begin{array}{ccc} \underset{T}{\underset{︸}{\left[\begin{array}{c} x_{1,1}\\ \vdots \\ x_{MNN,1} \end{array}\right.}} & \underset{SW}{\underset{︸}{\begin{array}{ccc} x_{1,2} & \cdots & x_{1,MNN+2}\\ \vdots & \ddots & \vdots \\ x_{MNN,2} & \cdots & x_{MNN,MNN+2} \end{array}}} & \underset{TF}{\underset{︸}{\left.\begin{array}{c} x_{1,MNN+3}\\ \vdots \\ x_{MNN,MNN+3} \end{array}\right]}} \end{array}. \end{array}$$

The matrix that represents the individual codifies three different types of information (topology, synaptic weights, and transfer function). In that sense, it is necessary to determine the exploring range of each type of information in its corresponding search space. For the case of the topology, the range is set between [1, 2^MNN^ − 1] due to the integer number of this part being codified into a binary vector composed of MNN elements that indicates if there is a connection between neuron *i* and neuron *j*.

The synaptic weights and bias have a range between [−4,4] and [−2,2] and for the transfer functions the range is [1, nF], where nF is the total number of transfer functions.

### 4.2. Architecture and Synaptic Weights

Once the individuals or possibles solutions are obtained, it is necessary to decode the matrix information **W** into an ANN for its evaluation. The first element to decode is the topology in terms of the synaptic weights and transfer functions that are stored in the matrix.

This research is limited to a kind of feed-forward ANN, for this reason some rules were proposed to guarantee that no recurrent connections will appear in the ANN (the unique restriction for the ANN). In future works, we will include recurrent connections and study the behavior of this type of ANNs.

The architectures generated by the proposed methodology will be composed of only three layers: input, hidden, and output. To generate valid architectures the following three rules must satisfied.

Let ILN be the set of *I* neurons composing the input layer, HLN the set of *J* neurons composing the hidden layer, and OLN the set of *K* neurons composing the output layer.

1. *For the input layer neurons (ILN),* the ILN_*i*_, *i* = 1,…, *I*, neuron only can send information to HLN_*j*_ and OLN_*k*_.
2. *For the hidden layer neurons (HLN),* the HLN_*j*_, *j* = 1,…, *J*, neuron only can send information to OLN_*k*_ and HLN_*j*_ with one restriction for the last. For HLN_*j*_ there is a connection only with HLN_*j*+1_,…, HLN_*J*_.
3. *For the output layer neuron (OLN),* the OLN_*k*_, *k* = 1,…, *K* neuron only can send information to other neurons of their layer but with a restriction, for OLN_*k*_ there is a connection only with OLN_*k*+1_,…, OLN_*K*_.

To decode the architecture taking into account these rules, the information in **W**_*ij*_ with *i* = 1,…, MNN and *j* = 1 (which is in decimal base) is codified based on the binary square matrix **Z**. This matrix will represent a graph where each component *z*_*ij*_ indicates the links between neuron *i* and neuron *j* when *z*_*ij*_ = 1. For example, suppose that **W**_*ij*_ has an integer number “57.” It is necessary to transform it into a binary code “0111001.” The binary code is interpreted as the connections of a *i*th neuron to seven neurons (number of bits). In this case, only neurons two, three, four, and seven (from left to right) links to neuron *i* are observed.

Then, the architecture is now evaluated with the corresponding synaptic weights of the component **W**_*ij*_ with *i* = 1,…, MNN and *j* = 2,…, MNN + 1. Finally, each neuron computes its output with its corresponding transfer function shown in the same array. In the case of bias, it is encoded in the component **W**_*ij*_ with *i* = 1,…, MNN and *j* = MNN + 2.

### 4.3. Transfer Functions

The TF are represented in the component **W**_*ij*_ with *i* = 1,…, MNN and *j* = MNN + 3. The transfer functions are in the range of [0,5] representing one of the six transfer functions selected in this work.

Although there are several transfer functions that can be used in the ANN context, in this work the most popular and useful transfer functions in several kinds of problems are selected. The transfer functions in this research with their labels to identify them are Sigmoid function (LS), hyperbolic tangent function (HT), sinusoidal function (SN), Gaussian function (GS), linear function (LN), and hard limit function (HL).

### 4.4. ANN Output

Once decoded the information from the individual is necessary to know its efficiency to be evaluated with any of the fitness functions. To do this, it is necessary to calculate the output of the ANN designed during the training stage and generalization stage. This output is calculated using Algorithm 2, where *o*_*i*_ is the output of the neuron *i*, *a*_*j*_ is the input pattern that feeds the ANN, *n* is the dimensionality of the input pattern, *m* is the dimensionality of the desired pattern, and *y*_*i*_ is the output of the ANN.

## 5. Proposed Fitness Functions

Each individual must be selected based on their fitness, and the best solution is taken depending on the evaluation (performance) of each individual. In this work, we propose eight different fitness functions to design an ANN. It is important to remark that fitness functions only are used during the training stage to evaluate each solution. After designing the ANN, we use a new metric that allows us to compare efficiently the results provided by the ANN generated with the proposed methodology.

### 5.1. Mean Square Error

The mean square error (MSE) represents the error between the ANN output and the desire patterns. In this case, the best individual is the one which generates the minimum MSE (see the following equation):

(16) $$\begin{array}{c} F_{1}=\text{MSE}=\left(\;\frac{1}{p\cdot M}\overset{p}{\underset{\xi =1}{\sum }}\;\overset{M}{\underset{i=1}{\sum }}{\left(\;d_{i}^{\xi }-y_{i}^{\xi }\;\right)}^{2}\;\right), \end{array}$$

where *y*_*i*_ is the output of the ANN.

### 5.2. Classification Error

The classification error (CER) is calculated as follows: the output of the ANN *y*_*i*_ is transformed into binary codification by means of the winner-take-all technique. The binary chain must have only a number 1 and the rest is composed of 0s. This indicates that the position with 1 is the class to which the input pattern belongs. This binary chain is compared against the desire pattern, if they are equal the classification was done correctly.

In this case, the best ANN is the one which generates the minimum wrong classified patterns. The CER is represented by

(17) $$\begin{array}{c} F_{2}=\text{CER}=\left(\;1-\frac{\text{npbc}}{\text{tpc}}\;\right), \end{array}$$

where npbc represents the number of patterns well classified and tpc is the total of patterns to classify.

### 5.3. Validation Error

When the ANN is trained during a long period, the ANN could get a maximum learning in which the ANN becomes adept (overfitting). However, this has a disadvantage because if the input data during the testing stage are contaminated with a negligible amount of noise, the ANN will not be able to recognize new patterns.

For that reason, we need to include a validation phase to prevent overfitting and thus guarantee an adequate generalization. Therefore, we designed a fitness function that integrates the assessment of both the training and validation stages.

Based on this idea, two fitness functions were generated: the first evaluates the mean square error (MSE) on the training set MSE_*T*_ and the MSE on the validation set MSE_*V*_; see (18). The second function takes into account both the classification error (CER) on the training set CER_*T*_ and the classification error on the validation set CER_*V*_; see (19):

(18) F3VMSE=0.6×MSEV+0.4×MSET,

(19) F4VCER=0.6×CERV+0.4×CERT.

In order to evaluate the fitness of each solution using (18) and (19), it is necessary to first computed the MSE or CER using the training set; after that, the MSE or CER using the validation set is computed. It is important to notice that the error achieved with the validation set is more weighted than the error obtained with the training set.

### 5.4. Reduction of the Architecture

In order to generate a smaller ANN in terms of the number of connections, it is necessary to design a fitness function that takes into account the performances of the ANN in terms of the MSE or CER as well as a factor related to the number of connections used in the ANN.

In that sense, we proposed the following equation for computing the factor that allows us to measure the size of the ANN in terms of the number of connections:

(20) $$\begin{array}{c} \text{RA}=\frac{\text{NC}}{\text{NMaxC}}, \end{array}$$

where NC represents the number of connections when the proposed methodology is applied and NMaxC represents the maximum number of connection that an ANN can generate which is computed as in

(21) $$\begin{array}{c} \text{NMaxC}=\overset{MNN}{\underset{i=n}{\sum }}i, \end{array}$$

where MNN is the maximum number of neurons.

It is important to mention that not necessarily less or more connections generate a better performance; however, by using factor RA, it is possible to weight other metrics that can measure the performance of the ANN and find the ANN with less connections with an acceptable performance.

In that sense, we proposed two new fitness functions in terms of the MSE function equation (22) and in terms of the CER function equation (23). These fitness functions tend to the global minimum when the factor RA and the performance are small; however, when one of these terms tends to increase, the fitness function tends to move away from the global minimum:

(22) F5RAMSE=RA·MSE,

(23) F6RACER=RA·CER.

### 5.5. Architecture Reduction and Validation Error with MSE and CER Errors

At last, two fitness functions RA*V*MSE and RA*V*CER were generated: the first reduces simultaneously the architecture, the validation error, and the MSE; see (24). The second function reduces the architecture, the validation error, and the CER equation (25):

(24) F7RAVMSE=RA·0.6×MSEV+0.4×MSET,

(25) F8RAVCER=RA·0.6×CERV+0.4×CERT.

## 6. Tuning the Parameters for PSO Algorithms

**Figure 2:**
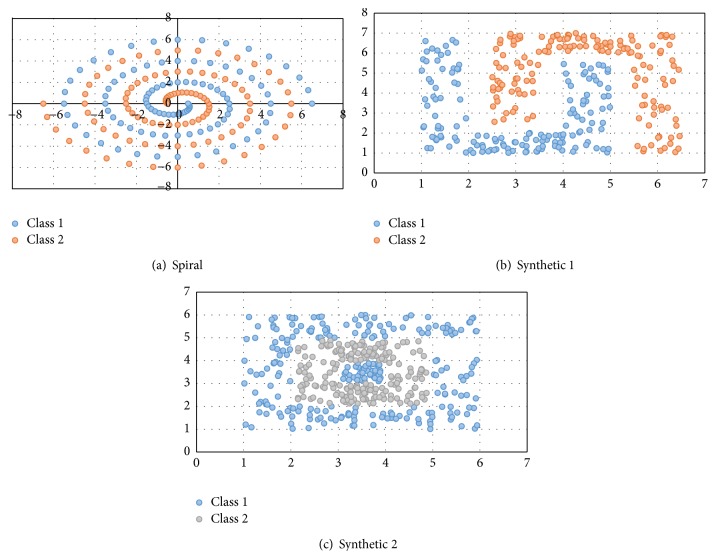
Pattern dispersion for the three synthetic problems. (a) Pattern dispersion for spiral problem. (b) Pattern dispersion for synthetic 1 problem. (c) Pattern dispersion for synthetic 2 problem.

Ten classification problems of different complexity were selected to evaluate the accuracy of the methodology: Iris plant, wine, breast cancer, diabetes, and liver disorder datasets which were taken from the UCI machine learning benchmark repository [25]. The object recognition problem was taken from [26], and the spiral, synthetic 1, and synthetic 2 datasets were developed in our laboratory. The pattern dispersions of these datasets are shown in Figure 2.

Table 1 shows the description for each classification problem.

Each dataset was randomly divided into three sets for training, testing, and validating the ANN as follows: 33% of the total patterns for the training stage, 33% for validation stage, and 34% for testing stage.

After that, the best parameter values for each algorithm were found to obtain the best performance for each classification problem. Then, the best configuration for each algorithm was used to validate statistically the accuracy of the ANN.

To determine which parameters generate the best ANN in terms of its accuracy, it is necessary to analyze training and testing performance. Although the accuracy of the ANN should be measured in terms of the testing performance, it is also important to consider the performance that achieves the ANN during the training stage, in order to find the parameters that provoke the best results during training and testing stages. Instead of analyzing the training and testing performances separately, we proposed a new metric that let us consider the accuracy of the ANN during training and testing stages. This metric allows us to weight the testing performance to validate the accuracy of the proposal and, at the same time, to have the confidence that training stage was done with an acceptable accuracy. This metric computed a weighted recognition rate (wrr) and it is described in

(26) $$\begin{array}{c} \text{wrr}=0.4\times \left(\;Tr_{r}r\;\right)+0.6\times \left(\;Te_{r}r\;\right), \end{array}$$

where Tr_r_r represents the recognition rate obtained during the training stage and Te_r_r represents the recognition rate obtained during the testing stage.

From (26), we could observe that testing and training stages were weighted by a factor of 0.6 and 0.4, respectively. Using these factors, we can avoid that high wrr value may be obtained by a higher training recognition rate and a lower testing recognition rate.

The analysis to select the best values of each algorithm was performed taking into account the ten classification problems described above. The different parameters for each algorithm were varied in different ranges to evaluate the performance of the algorithms over different pattern recognition problem. In order to find the best configuration for the parameters of each algorithm, several experiments were done assigning different values to each parameter in the three bioinspired algorithms (original PSO, SGPSO, and NMPSO).

The parameters were divided into two types: the parameters that are shared or common to all algorithms, such as the number of generations, the number of individuals, the range of variables, and the fitness function. The specific parameters are those that are unique or specific to each algorithm, for example, for the basic PSO algorithm, inertia *ω* and the two coefficients of acceleration *c*_1_ and *c*_2_ are the parameters that change. In the case of SGPSO algorithm takes two parameters, the coefficient of acceleration *C*_3_ and the geometric center $\bar{P}$. Finally, the NMPSO algorithm has the crossover operator *α*, the mutation operator *β*, and *γ* which determine when each neighborhood should be updated.

For each parameter configuration and each problem 5 experiments with 2000 generations were performed. Once the ANNs were designed with the proposed methodology, the average weighted recognition rate wrr was obtained.

Next is described which values were taken for each parameter to obtain the best configuration for each bioinspired algorithm.

The common parameters for the three algorithms are represented as follows: for the population size, in the variable *v* = {50,100} the first element corresponds to 50 individuals and the second corresponds to 100 individuals. In the case of the search space size *w* = {2,4} the first element indicates that the range is set to [−2,2] and the second item indicates that the range is between [−4,4]. The type of fitness function used with the bioinspired algorithm is represented by the variable *x* and can take one of the eight elements, *x* = {MSE, CER, *V*MSE, *V*CER, RAMSE, RACER, RA*V*MSE, RA*V*CER}.

All the possible combinations using the different parameter values were tested. The eighth fitness function was tested using all the classification problems proposed in this research to see which provides the best accuracy.

The configuration to determine the value for each parameter for original PSO is determined by the following sequence: *v* − *w* − *x* − *u* − *y* − *z*.

The basic PSO algorithm has three unique parameters: the inertia weight *ω* represented by *u* which can take the following values *u* = 0.3,0.5,0.7,0.9 and the two acceleration coefficients *c*_1_ and *c*_2_ represented by *y* and *z*, respectively, with the values *y* = *z* = 0.5,1.0,1.5. Once we finished the set of experiments to test the performance of the original (basic) PSO algorithm with all the previous values combinations, we found that the best parameter configuration was 100 − [−2,2] − *V*CER − 0.3 − 1.0 − 1.5.

SGPSO algorithm has two unique parameters: the acceleration coefficient *C*_3_ represented by the variable *y* whose values are *y* = 0.5,1.0,1.5 and the geometric center $\bar{P}$ represented by the variable *z* with values *z* = 100,200,300. In the case of the acceleration coefficients *c*_1_ and *c*_2_, it took the best values found for the basic PSO algorithms: *c*_1_ = 1.0, *c*_2_ = 1.5, and *ω* = 0.3. After several experiments, the best parameter configuration for SGPSO was 100 − [−2,2] − CER − 0.5 − 100.

The NMPSO algorithm has three unique parameters: the updating neighborhood rate *γ* which takes the values *u* = 100,200,300, the crossover factor *α*, and the mutation factor *β*, which are represented by the variables *y* and *z*; both take the values *y* = *z* = 0.1,0.5,0.9. The best parameter configuration found for NMPSO was 100 − [−4,4] − CER − 200 − 0.1 − 0.1.

## 7. Experimental Results

Once we determined the best configuration for each algorithm, we performed an exhaustive testing of 30 runs for each pattern classification problem. The accuracy of the ANN generated by the methodology was measured in terms of the weighted recognition rate (26). The following subsections describe the results obtained for each database and each bioinspired algorithm. These experiments show the evolution of the fitness function during 5000 generations, the weighted recognition rate, and some examples of the architectures generated with the methodology.

### 7.1. Results for Basic PSO Algorithm

**Figure 3:**
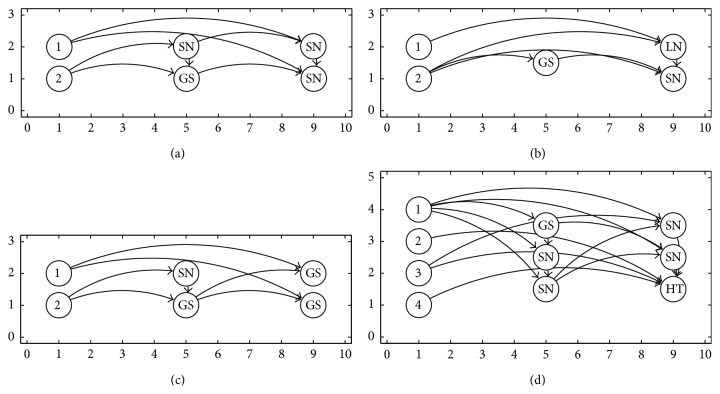
Some ANN generated using basic PSO algorithm. (a) The best architecture for spiral problem. (b) The best architecture for synthetic 1 problem. (c) The best architecture for synthetic 2 problem. (d) The best architecture for Iris plant problem.

In Figure 3 are shown some of the ANNs generated using the PSO algorithm that provide the best results for the recognition problem.

**Figure 4:**
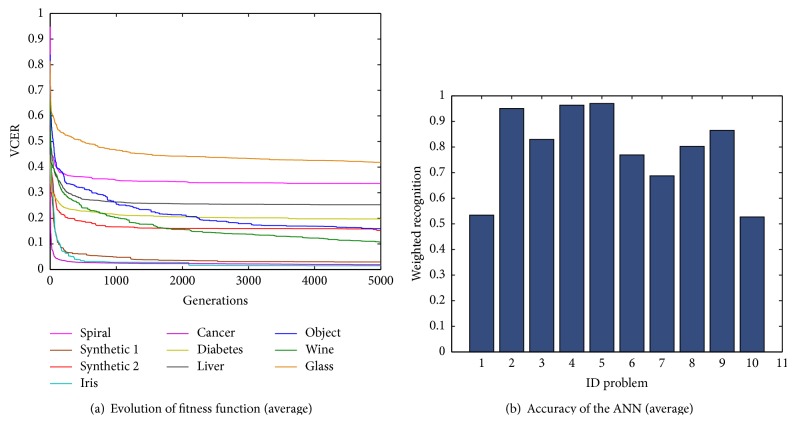
Average results for ten classification problems using basic PSO algorithm. (a) Average error evolution. (b) Average weighted recognition percentage.

Figure 4(a) showed the evolution of the fitness function *V*CER where we can appreciate the tendency for each classification problem. These results were obtained with the best configuration of basic PSO.

The evolution of the fitness function represents the average of the 30 experiments for each problem. It is observed that the value of the fitness function for the glass, spiral, liver disorders, diabetes, and synthetic 2 problems slightly decreases despite the number of generations. Smaller values for the fitness function were achieved with the Iris plant, breast cancer, and synthetic 1 problems. With the object recognition and wine problems, the value of the fitness function decreased when approaching the limit of generations. The average weighted recognition rate for each problem is presented in Figure 4(b). It can be observed that, for the glass problem, the ANN achieved the smallest average weighted recognition rate (52.67%), followed by the spiral (53.39%), liver disorders (68.74%), diabetes (76.90%), object recognition (80.22%), synthetic 2 (82.96%), and wine (86.49%). The highest average weighted recognition rates were achieved for the synthetic 1 (95.03%), the Iris (96.35%), and the breast cancer (96.99%).

Table 2 presents the frequency at which the six different transfer functions were selected for the ANN during the training stage. Applying the PSO algorithm, we see that there is a small range of selected functions. For example, the sinusoidal function was selected more often for the spiral, synthetic 1, and synthetic 2 problems. The Gaussian transfer function was selected more often for Iris plant, breast cancer, diabetes, liver disorders, object recognition, wine, and glass problems.

Table 3 shows the maximum, minimum, standard deviation, and average number of connections used by the ANN. As you can see, in average, the number of connections is low for the problems of spiral, synthetic 1, and synthetic 2. For the glass and wine, in average, 97.43 and 91.1 connections were used, respectively.

Table 4 shows the maximum, minimum, standard deviation, and average the number of neurons used in the ANN generated with the proposed method. In this table, we can see that the number of neurons in the ANN for the ten classification problems was no more than 13.

### 7.2. Results for SGPSO Algorithm

**Figure 5:**
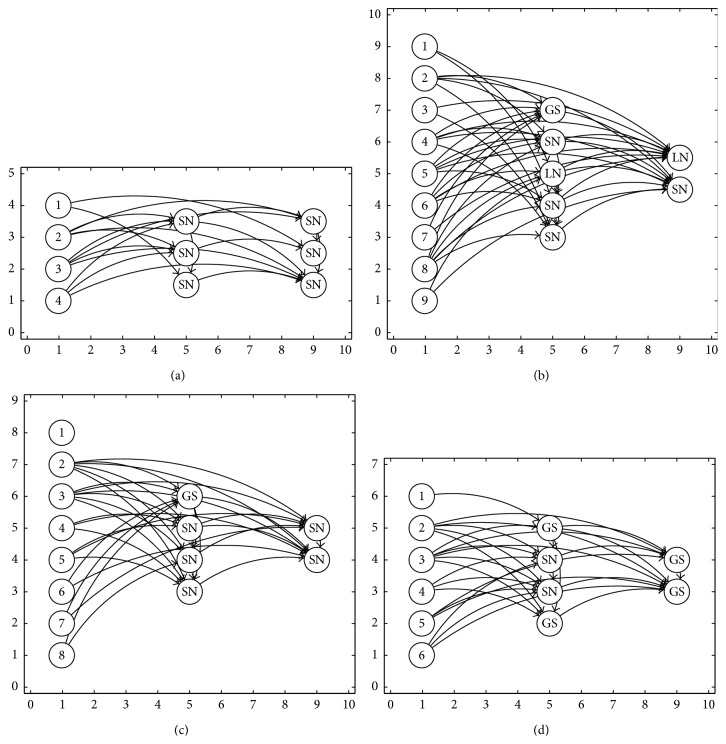
Some ANN using SGPSO algorithm. (a) The best architecture for Iris plant problem. (b) The best architecture for breast cancer problem. (c) The best architecture for diabetes problem. (d) The best architecture for liver disorders problem.

In Figure 5 are shown some of the best ANNs generated with the SGPSO algorithm. You can also observe an example of an ANN with a input neuron without any connection; see Figure 5(c). The lack of connection in the ANN indicates that the input feature was not necessary to solve the problem. In other words, a dimensionality reduction of the input pattern was also done by the proposed methodology.

**Figure 6:**
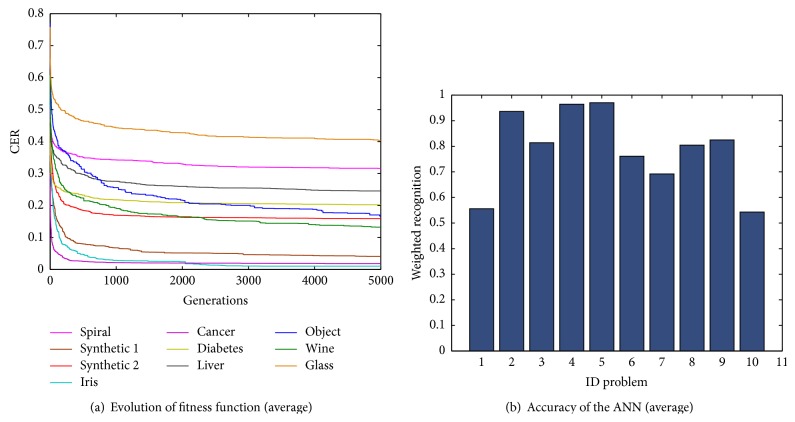
Average results for ten classification problems using basic SGPSO algorithm. (a) Average error evolution. (b) Average weighted recognition rate.

Figure 6(a) shows the evolution of the fitness function CER where we can see the tendency of the fitness function for each classification problem. These results were obtained with the best parameter configuration for the SGPSO algorithm. In general, the problems whose values are near to the optimal solution are the breast cancer, Iris plant, and synthetic 1, being in last place with high errors the liver disorders, glass, and spiral problems.

The average weighted recognition rate for each problem is presented in Figure 6(b). It was observed that for the glass problem the proposed methodology achieved the smallest weighted recognition rate (54.31%), followed by the spiral (55.60%), liver disorders (69.19%), diabetes (76.09%), object recognition (80.45%), synthetic 2 (81.39%), wine (82.47%), and synthetic 1 (93.61%). The second highest weighted recognition rate was achieved for the Iris plant (96.45%). The highest weighted recognition rate was achieved for the breast cancer problem (97.03%).

Table 5 presents the number of times that transfer functions were selected using the SGPSO algorithm. The sinusoid function was the most selected by 9 of the 10 classification problems: spiral, synthetic 1 and synthetic 2, Iris plant, diabetes, liver disorders, object recognition, wine, and glass problems. For the breast cancer problem, sinusoid function was selected almost at the same rate as the Gaussian function.

Furthermore, Table 6 shows the maximum, minimum, standard deviation, and average number of connections used by the ANN designed with the proposed methodology. In this case, SGPSO generates more connections between neurons of the ANN for the ten classification problems than those generated with the basic PSO algorithm.

Table 7 shows the maximum, minimum, standard deviation, and average number of neurons required for the ANN using SGPSO algorithm.

### 7.3. Results for NMPSO Algorithm

**Figure 7:**
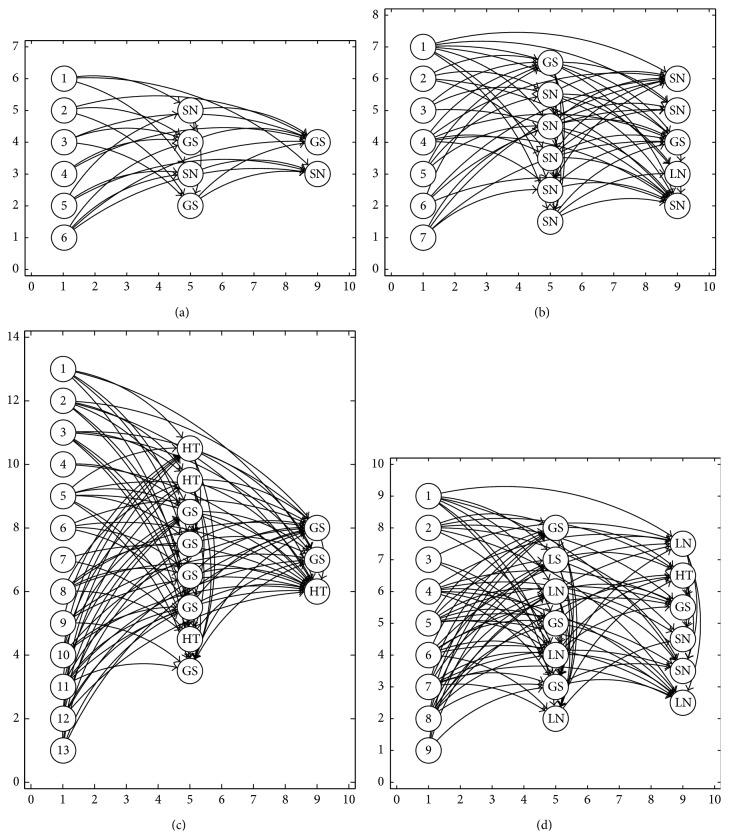
Some ANN using NMPSO algorithm. (a) The best architecture for liver disorders problem. (b) The best architecture for object recognition problem. (c) The best architecture for wine problem. (d) The best architecture for glass problem.

Figure 7 shows some of the best ANNs generated with the NMPSO algorithm. The fitness function used with the NMPSO algorithm was CER function.

**Figure 8:**
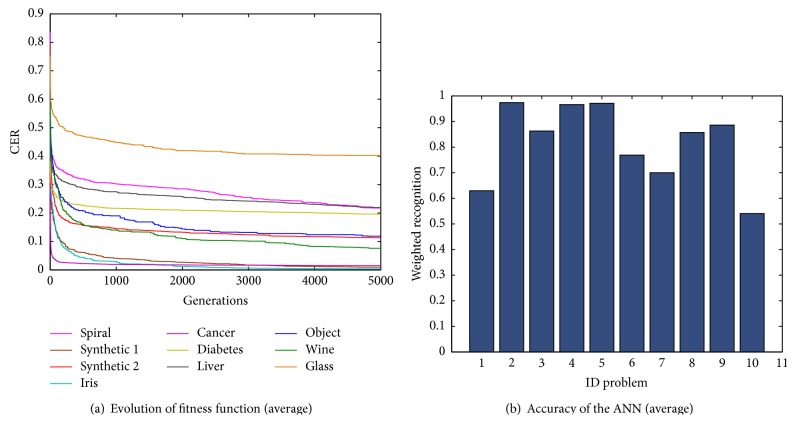
Average results for ten classification problems using NMPSO algorithm. (a) Average error evolution. (b) Average weighted recognition rate.

The evolution of the fitness function for the 10 classification problems is shown in Figure 8(a) where it is observed that the minimum values are reached with the synthetic 1, breast cancer, and Iris plant problems. For the case of wine problem the value of the fitness function improves while the generation's number increased. The worst case was observed for the glass problem.

The weighted recognition rate for each problem is shown in Figure 8(b). From this graph, we observed that the average weighted recognition rate for the glass problem was 54.06%, for the spiral problem 62.97% and for liver disorders it achieved 70.01%, the diabetes problem 76.89%, the object recognition problem 85.73%, and synthetic problem 2 86.30%. The best recognition rate was achieved with the wine problem (88.62%), Iris plant (96.60%), breast cancer (97.11%), and synthetic 1 (97.42%).

The number of times that the transfer functions were selected using NMPSO algorithm is described in Table 8. Using the sinusoidal function, the ANNs provide better results for the spiral, synthetic problem 1, synthetic problem 2, and the object recognition problem. For the the Iris plant, breast cancer, diabetes, liver disorders, wine, and glass problems the Gaussian function was the most selected.

In general, the transfer function most often selected using NMPSO algorithm was the Gaussian, second sinusoidal function, then the hyperbolic tangent, next the linear function, and the last places the sigmoid and hard limit functions.  Table 9 shows the maximum, minimum, standard deviation, and average connections number.

In Table 10 are shown the maximum, minimum, standard deviation, and the average number of neurons used by the ANN generated with the NMPSO algorithm.

## 8. General Discussion

In general, Table 11 shows a summary of results taking into account the average weighted recognition rate obtained with the three bioinspired algorithms.

For the cases of the spiral, synthetic 1, Iris plant, breast cancer, liver disorders, object recognition, and wine problems the algorithm providing better results was the NMPSO algorithm. For the glass problem the best accuracy was achieved with SGPSO algorithm and for the case of diabetes the best performance was achieved using the basic PSO algorithm.

From Table 11, it is possible to see that the best algorithm, in terms of the weighted recognition rate, was NMPSO (81.57%), the second best algorithm was basic PSO (78.97%), and the last was SGPSO algorithm (78.65%) for the ten classification problems.

Moreover, these results were compared with results obtained from classic algorithms such as the gradient descent and Levenberg-Marquardt. Due to the classic techniques needing a specific architecture, it was proposed to design manually two kinds of ANN. The first consists of one hidden layer and the second consists of two hidden layers.

To determine the maximum number of neurons MNN used to generate the ANN we follow the same rule proposed in the methodology. For the ANN with two hidden layers, there was a pyramidal distribution using

(27) $$\begin{array}{c} \text{DN}=0.6\times \left(\;\text{MNN}\;\right)+0.4\times \left(\;\text{MNN}\;\right), \end{array}$$

where the first hidden layer has the 60% of the total hidden layers and the second hidden layer has the 40% of the total hidden layers.

Two stop criteria for the gradient descent and Levenberg-Marquardt algorithms were established: until the algorithm reach 5000 epochs or until reach an error of 0.000001. The classification problems were divided into three subsets: 40% of the overall patterns were used for training, 50% for generalization, and 10% for validation. The learning rate was set to 0.1.

In Table 12 is shown the average weighted recognition rate using the classic training algorithms: one based on gradient descent (backpropagation algorithm) and the other based on the Levenberg-Marquardt algorithm. From this set of experiments, we observed that the best algorithm was Levenberg-Marquardt with a single layer. This algorithm solved eight of ten problems with the best performance (spiral, synthetic 1, synthetic 2, Iris plant, breast cancer, diabetes, liver disorders, and object recognition). For the case of the wine problem, the best algorithm was the gradient descent algorithm composed of one single layer. The glass problem was solved better using Levenberg-Marquardt with two hidden layers.

Considering Tables 12 and 11, the best techniques to design ANN were the NMPSO algorithm followed by the Levenberg-Marquardt with one hidden layer. On the other hand, the basic PSO and SGPSO algorithms as well as the gradient descend and Levenberg-Marquardt with two layers did not provide a good performance.

Besides that Levenberg-Marquardt obtained better results than PSO and SGPSO algorithms, there are some important points to consider: first, the ANN designed with the proposed methodology includes the selection of the architecture, synaptic weights, bias, and transfer functions. For the case of classic techniques, the architectures must be carefully and manually designed by an expert in order to obtain the best results; this process can be a time-consuming task for the expert. On the opposite side, the proposed methodology automatically designs the ANN in terms of the input and desire patterns that codified the problem to be solved.

## 9. Conclusions

In this paper, we proposed three connection rules for generating feed-forward ANN and guiding the connections between neurons. These rules allow connections among neurons from the input layer to the output layer. These rules also allow to generate lateral connections among neurons from the same layer.

We also observed that some ANNs designed by the proposed methodology do not have any connection from the input neurons. It means that the feature associated to this neuron was not relevant to compute the output of ANN. This is known as dimensionality reduction of the input pattern.

Eight transfer functions, which involve the combination of the MSE, CER validation error, and architecture reduction (of connections and neurons), were implemented to evaluate each individual. From these experiments, we observed that the fitness functions that generated the ANN with the best weighted recognition rate were those that used the classification error CER. The three bioinspired algorithms based on PSO were compared in terms of the average weighted recognition rate.

On the other hand, the NMPSO algorithm achieved the best performance followed by the basic PSO and SGPSO algorithm.

To validate statistically the accuracy of the proposed methodology, first of all, the parameters for the three bioinspired algorithms were selected. For the case of basic PSO the best fitness function selected was *V*CER with a variable range between [−2,2]. After tuning the parameters of each algorithm and choosing the best configuration, we observe that the parameters were different from those proposed in the literature; these values for the parameters were set to *ω* = 0.3, *c*_1_ = 1.0, and *c*_2_ = 1.5. For the SGPSO algorithm, the best fitness function selected was CER with a variable range between [−2,2]. The values for the parameters were set to *c*_3_ = 0.5 and the geometric centre $\bar{P}=100$. For the NMPSO algorithm, the best fitness function was CER with a variable range between [−4,4]. The parameters for the best configuration were set to *γ* = 200, crossover rate *α* = 0.1, and mutation rate *β* = 0.1.

After tuning the parameters of the three algorithms, 30 runs were performed for each of the ten classification problems. In general, whereas the problems that achieved a weighted recognition rate of 100% were the synthetic problem 1, Iris plant, and object recognition problems, a lower performance was obtained with the glass and spiral problems.

The transfer functions that more often were selected for each algorithm were: the Gaussian function for the basic PSO algorithm, the sinusoidal function for SGPSO algorithm and the Gaussian function for NMPSO algorithm.

In general, the ANNs designed with the proposed methodology were very promising. The proposed methodology automatically designs the ANN based on determining the set connections, the number of neurons in hidden layers, the adjustment of the synaptic weights, the selection of bias, and transfer function for each neuron.

## Figures and Tables

**Algorithm 1:**
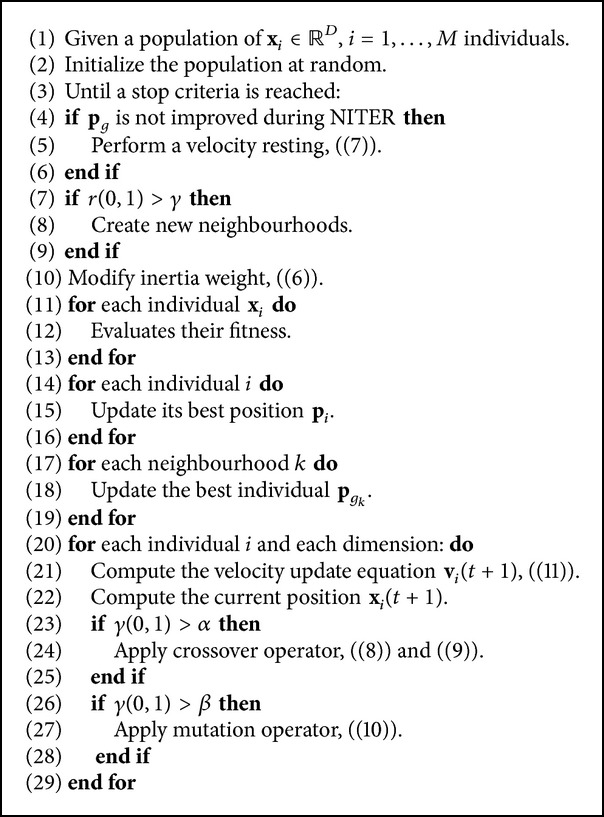
New Model of PSO pseudocode.

**Algorithm 2:**
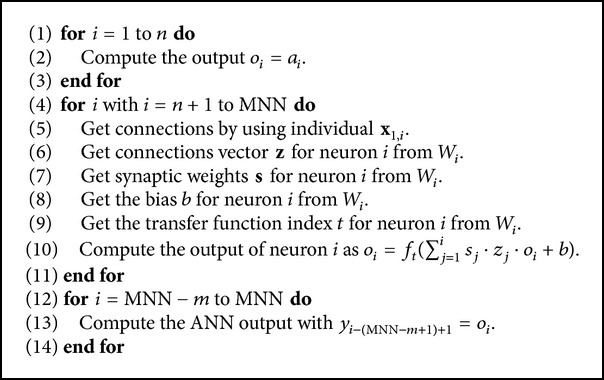
Output of the ANN pseudocode.

**Table 1:** Classification problems description.

ID problem	Classification problems	Pattern description	Total patterns
1	Spiral	2 characteristics that describe 2 classes	194
2	Synthetic 1	2 characteristics that describe 2 classes	300
3	Synthetic 2	2 characteristics that describe 2 classes	450
4	Iris plant	4 characteristics that describe 3 classes	150
5	Breast cancer	9 characteristics that describe 2 classes	683
6	Diabetes	8 characteristics that describe 2 classes	768
7	Liver disorders	6 characteristics that describe 2 classes	345
8	Object recognition	7 characteristics that describe 5 classes	100
9	Wine	13 characteristics that describe 3 classes	178
10	Glass	9 characteristics that describe 6 classes	214

**Table 2:** Number of times that transfer function was selected by basic PSO algorithm.

ID problem	Classification problems	Sigmoid function (LS)	Hyp. Tan. function (HT)	Sinusoid function (SN)	Gaussian function (GS)	Linear function (LN)	Hard limit function (HL)
1	Spiral	3	3	66	32	9	0
2	Synthetic 1	1	10	51	29	18	2
3	Synthetic 1	2	12	57	29	9	7
4	Iris plant	6	27	51	63	25	1
5	Breast cancer	2	40	51	61	32	8
6	Diabetes	4	30	57	76	26	0
7	Liver disorders	3	18	62	65	19	4
8	Object recognition	6	33	116	126	40	6
9	Wine	1	46	98	125	42	3
10	Glass	5	54	140	145	46	0

	Total	33	273	749	**751**	266	31

**Table 3:** Number of connections used by the ANN generated with the basic PSO algorithm.

ID problem	Classification problems	Connection number			
		Minimum	Maximum	Average	Std. dev.
1	Spiral	4	10	7.2667	1.4126
2	Synthetic 1	5	10	7.0667	1.2576
3	Synthetic 2	5	9	7.5	1.3326
4	Iris plant	14	23	18.9667	2.4563
5	Breast cancer	19	50	38	6.9926
6	Diabetes	8	47	35	7.0759
7	Liver disorders	16	31	24.6667	3.8177
8	Object recognition	54	71	62.8	4.9578
9	Wine	59	109	91.1	13.2336
10	Glass	81	108	97.4333	6.3933

**Table 4:** Number of neurons used by the ANN generated with the basic PSO algorithm.

ID problem	Classification problems	Neurons number			
		Minimum	Maximum	Average	Std. dev.
1	Spiral	3	4	3.7667	0.4302
2	Synthetic 1	2	4	3.7	0.5350
3	Synthetic 2	3	4	3.8667	0.3457
4	Iris plant	5	6	5.7667	0.4302
5	Breast cancer	3	7	6.4667	0.9371
6	Diabetes	2	7	6.4333	1.0063
7	Liver disorders	4	6	5.7	0.5350
8	Object recognition	10	11	10.9	0.3051
9	Wine	8	11	10.5	0.8610
10	Glass	13	13	13	0

**Table 5:** Number of times that transfer function was selected by SGPSO algorithm.

ID problem	Classification problems	Sigmoid function (LS)	Hyp. Tan. function (HT)	Sinusoid function (SN)	Gaussian function (GS)	Linear function (LN)	Hard limit function (HL)
1	Spiral	0	9	70	30	1	0
2	Synthetic 1	0	2	72	38	2	0
3	Synthetic 2	0	1	80	29	2	0
4	Iris plant	2	13	103	54	4	0
5	Breast cancer	5	28	71	72	22	1
6	Diabetes	2	17	93	73	6	0
7	Liver disorders	2	12	99	56	2	0
8	Object recognition	0	11	198	116	3	0
9	Wine	3	34	134	120	24	1
10	Glass	0	24	215	144	4	0

	Total	14	151	**1135**	732	70	2

**Table 6:** Number of connections used by the ANN generated with the SGPSO algorithm.

ID problem	Classification problems	Connection number			
		Minimum	Maximum	Average	Std. dev.
1	Spiral	4	10	6.8667	1.8520
2	Synthetic 1	4	9	6.6333	1.4016
3	Synthetic 2	5	10	7.0667	1.4368
4	Iris plant	12	25	19.7667	3.025
5	Breast cancer	30	49	40.9333	4.2906
6	Diabetes	20	47	36.7	6.8337
7	Liver disorders	14	34	26.9333	4.1517
8	Object recognition	52	79	66.1667	6.2648
9	Wine	71	107	93.833	9.0443
10	Glass	81	109	96.2	7.4575

**Table 7:** Number of neurons used by the ANN generated with the SGPSO algorithm.

ID problem	Classification problems	Neurons number			
		Minimum	Maximum	Average	Std. dev.
1	Spiral	2	4	3.6667	0.5467
2	Synthetic 1	3	4	3.8	0.4068
3	Synthetic 2	3	4	3.7333	0.4498
4	Iris plant	4	6	5.8667	0.4342
5	Breast cancer	5	7	6.6333	0.5561
6	Diabetes	4	7	6.3667	0.8899
7	Liver disorders	4	6	5.7	0.5350
8	Object recognition	10	11	10.9333	0.2537
9	Wine	9	11	10.5333	0.6288
10	Glass	12	13	12.9	0.3051

**Table 8:** Number of times that transfer function was selected by NMPSO algorithm.

ID problem	Classification problems	Sigmoid function (LS)	Hyp. Tan. function (HT)	Sinusoid function (SN)	Gaussian function (GS)	Linear function (LN)	Hard limit function (HL)
1	Spiral	0	1	80	25	10	0
2	Synthetic 1	1	9	48	44	6	1
3	Synthetic 2	0	3	62	41	6	1
4	Iris plant	5	17	63	68	17	3
5	Breast cancer	2	41	52	62	29	3
6	Diabetes	3	24	55	82	23	1
7	Liver disorders	0	9	65	81	4	0
8	Object recognition	0	19	174	115	19	0
9	Wine	3	58	76	137	40	5
10	Glass	1	53	114	172	48	2

	Total	15	234	789	**827**	202	16

**Table 9:** Number of connections used by the ANN generated with the NMPSO algorithm.

ID problem	Classification problem	Connections number			
		Minimum	Maximum	Average	Std. dev.
1	Spiral	4	10	7.1333	1.4320
2	Synthetic 1	3	10	6.5333	1.4559
3	Synthetic 2	4	10	7.4667	1.5916
4	Iris plant	15	24	19.2667	2.6121
5	Breast cancer	22	51	38.6333	7.7792
6	Diabetes	12	44	34.5667	7.6775
7	Liver disorders	16	30	22.8667	4.9461
8	Object recognition	51	70	62.8667	5.3223
9	Wine	76	109	92.9333	9.1007
10	Glass	79	107	95.4333	6.8213

**Table 10:** Number of neurons used by the ANN generated with the SGPSO algorithm.

ID problem	Classification problem	Connections number			
		Minimum	Maximum	Average	Std. dev.
1	Spiral	3	4	3.8667	0.3457
2	Synthetic 1	2	4	3.6333	0.5561
3	Synthetic 2	3	4	3.7667	0.4302
4	Iris plant	5	6	5.7667	0.4302
5	Breast cancer	4	7	6.3	0.9154
6	Diabetes	3	7	6.2667	0.9803
7	Liver disorders	4	6	5.3	0.7944
8	Object recognition	10	11	10.9	0.3051
9	Wine	9	11	10.6333	0.6149
10	Glass	13	13	13	0

**Table 11:** Average weighted recognition rate (wrr) for the three bioinspired algorithms.

Classification problems	Basic PSO algorithm	SGPSO algorithm	NMPSO algorithm
Spiral	0.53389	0.55601	**0.62969**
Synthetic 1	0.95033	0.93608	**0.97417**
Synthetic 2	0.82955	0.81386	**0.86299**
Iris plant	0.96346	0.96453	**0.96604**
Breast cancer	0.96993	0.9703	**0.97113**
Diabetes	**0.76895**	0.76092	0.76885
Liver disorders	0.68736	0.69187	**0.70007**
Object recognition	0.80222	0.80453	**0.85733**
Wine	0.86485	0.82471	**0.88621**
Glass	**0.52666**	0.54305	0.54062

Average wrr	0.78972	0.78658	**0.81571**

**Table 12:** Average weighted recognition rate (wrr) for the classic algorithms.

Classification problems	Descent gradient (one layer)	Descent gradient (two layers)	Levenberg-Marquardt (one layer)	Levenberg-Marquardt (two layers)
Spiral	0.50082	0.50137	**0.5092**	0.50137
Synthetic 1	0.74991	0.77044	**0.79008**	0.77728
Synthetic 2	0.54485	0.51442	**0.69997**	0.56248
Iris plant	0.93226	0.65226	**0.97911**	0.75626
Breast cancer	0.96769	0.94475	**0.96926**	0.95741
Diabetes	0.75786	0.7276	**0.76526**	0.7609
Liver disorders	0.60443	0.57651	**0.67561**	0.66258
Object recognition	0.74453	0.69413	**0.98213**	0.72746
Wine	**0.98292**	0.93378	0.96861	0.9791
Glass	0.70704	0.68535	0.78903	**0.79838**

Average wrr	0.74923	0.70006	**0.81283**	0.74832
